# Longitudinal Seasonal Development of Anthropometric and Physical Performance Characteristics in Youth Trained Soccer Players

**DOI:** 10.3390/sports14070308

**Published:** 2026-07-20

**Authors:** Mohamed Amin Selmi, Roland van den Tillaar, Radhouane Haj Sassi, Raouf Hammami

**Affiliations:** 1 Higher Institute of Sport and Physical Education of Ksar-Said, University of Manouba, University Campus, Manouba 2010, Tunisia; selmi.med.amin@gmail.com (M.A.S.); raouf.cnmss@gmail.com (R.H.); 2 Tunisian Research Laboratory ‘Sports Performance Optimization’, National Center of Medicine and Science in Sports (CNMSS), CNMSS-LR09SEP01, Tunis 1004, Tunisia; 3 Department of Sport Sciences and Physical Education, Nord University, Høgskoleveien 27, 7600 Levanger, Norway; 4 Higher Institute of Sport and Physical Education of Kef, University of Jendouba, University Campus, Kef 8100, Tunisia

**Keywords:** football, seasonal variation, maximal aerobic speed, jump performance, maximal sprinting speed, repeated-sprint sets ability, anthropometrics

## Abstract

This study investigated seasonal changes in anthropometric characteristics, vertical and horizontal jump performance, 30 m sprint performance, estimated maximal aerobic capacity, and repeated-sprint ability in competitive youth soccer players across the preparation, mid-season, and end-season phases. A longitudinal repeated-measures design was employed over one competitive season. Anthropometric variables, vertical and horizontal jump performance, 30 m sprint performance, estimated VO_2_max derived from the Vam-Eval field test, and repeated-sprint sets (2 × 5 × 20 m with 15 s recovery between sprints and 1 min between sets) were assessed at three time points. Significant seasonal changes were observed in anthropometric characteristics, with body mass and height increasing progressively and body fat percentage decreasing throughout the competitive season (F ≥ 12.0, *p* < 0.001, η_p_^2^ ≥ 0.32). Body mass increased from 64.1 ± 6.7 to 66.7 ± 6.8 kg, height from 1.69 ± 0.07 to 1.72 ± 0.07 m, and body fat percentage decreased from 11.3 ± 2.0% to 9.4 ± 1.8%. Peak height velocity (PHV) significantly influenced changes in body mass and height but not body fat percentage. Seasonal improvements were observed in several physical performance measures. Squat jump, standing long jump, 15 s repeated jump performance, 10- and 30 m sprint performance, estimated maximal aerobic speed, estimated VO_2_max, and repeated-sprint performance changed significantly across the season (F ≥ 3.28, *p* ≤ 0.046, η_p_^2^ ≥ 0.11), whereas countermovement jump, five-jump test, and flying 20 m sprint performance remained unchanged. Estimated VO_2_max and maximal aerobic speed improved from preparation to mid-season and were subsequently maintained. Repeated-sprint performance exhibited set-dependent seasonal adaptations, with significant Time × Set interactions indicating progressive improvements during the second repeated-sprint set, whereas the first set showed only transient improvements at mid-season. Significant PHV and Time × PHV effects were also observed for aerobic capacity and repeated-sprint performance. In conclusion, these seasonal variations likely reflect the combined influence of biological maturation, training exposure, and competitive demands rather than training effects alone.

## 1. Introduction

Soccer is a high-intensity intermittent sport that requires players to repeatedly perform explosive actions such as sprinting, jumping, accelerating, decelerating, and changing direction while simultaneously maintaining a high level of aerobic fitness throughout a match [1,2]. The ability to sustain these physical demands is determined by a combination of anthropometric characteristics, neuromuscular power, speed, aerobic fitness, and repeated-sprint [3,4]. Consequently, monitoring the development of these performance-related variables throughout a competitive season is essential for understanding training adaptations and optimizing long-term athlete development [5].

The competitive season is characterized by fluctuations in training loads, match exposure, recovery opportunities, and physiological stress, which may influence physical performance [6]. Previous studies have shown that structured pre-season training can improve aerobic capacity, sprint performance, and muscular power [7,8]. However, the extent to which these adaptations are maintained throughout the competitive season remains unclear, with some studies reporting stable performance levels [9], whereas others have observed declines in sprint ability, jump performance, and fatigue resistance during later phases of competition [10,11]. These discrepancies highlight the need for longitudinal investigations examining multiple physical fitness components across different stages of the season. In youth soccer players, this interpretation is further complicated by ongoing growth and maturation, as increases in body mass, stature, and changes in body composition may occur simultaneously with training-related adaptations and independently influence physical performance outcomes [12,13]. Although previous research has examined seasonal changes in individual fitness components or anthropometric characteristics [14,15], fewer studies have simultaneously investigated growth-related changes, explosive power, sprint performance, aerobic fitness, and repeated-sprint ability within the same cohort, limiting our understanding of how maturation and competitive exposure interact over a full season.

Repeated-sprint ability is particularly important in soccer because many decisive match actions occur during repeated high-intensity efforts separated by short recovery periods [16,17,18]. Although widely investigated, most studies have assessed repeated-sprint performance using a single protocol, which may not fully replicate the demands of competitive match play [3]. Recent evidence suggests that evaluating performance across successive repeated-sprint sets may provide additional insight into fatigue resistance and recovery capacity under conditions that better reflect the intermittent nature of soccer [19,20]. Nevertheless, longitudinal studies examining seasonal changes in repeated-sprint performance across multiple sets remain limited.

Therefore, comprehensive longitudinal studies incorporating multiple testing points and a broad range of performance indicators are needed to better characterize seasonal changes in youth soccer players. The present study aimed to examine seasonal variations in anthropometric characteristics, jump performance, sprint performance, estimated maximal aerobic capacity, and repeated-sprint ability across the preparation, mid-season, and end-season phases of a competitive season. Additionally, repeated-sprint performance was evaluated across two consecutive sets to provide a more detailed assessment of fatigue resistance and set-dependent responses [21]. The novelty of this study lies in the integrated assessment of maturation-related anthropometric development and multiple physical performance determinants, including set-specific repeated-sprint responses, throughout an entire competitive season.

Therefore, the objective of this study was to examine seasonal changes in anthropometric characteristics, vertical and horizontal jump performance, sprint performance, estimated maximal aerobic capacity derived from the Vam-Eval field test, and repeated-sprint ability across the preparation, mid-season, and end-season phases in youth soccer players. Based on previous longitudinal studies [7,14,15], it was hypothesized that body mass and height would increase, whereas body fat percentage would decrease throughout the season. Furthermore, jump and sprint and maximal aerobic capacity performances were expected to improve from preparation to mid-season following training adaptations [22]. Consistent with evidence of accumulated fatigue during the competitive season [23], these performance gains were expected to partially decline toward the end of the season while remaining above baseline levels [7,14,15]. Finally, repeated-sprint performance was hypothesized to improve during the season, with lower performance observed in the second sprint sets due to fatigue accumulation [10,24].

## 2. Materials and Methods

### 2.1. Study Design

A longitudinal repeated-measures design was employed to investigate seasonal changes in anthropometric characteristics, jump performance, sprint performance, estimated maximal aerobic capacity, and repeated-sprint ability in competitive youth soccer players. Assessments were conducted at three time points during the competitive season: at the end of the preparation period, mid-season, and end-season. This longitudinal approach is commonly used to examine temporal variations in physical performance and the combined effects of training exposure, maturation, and competitive demands in youth soccer players [25].

### 2.2. Participants

A total of 27 competitive youth soccer players voluntarily participated in this study and completed all testing sessions throughout the competitive season. At baseline (preparation phase), players presented the following anthropometric characteristics: age, 14.6 ± 0.2 years; body mass, 64.1 ± 6.7 kg; height, 1.69 ± 0.07 m; and body fat percentage, 11.3 ± 2.0%. The predicted years from peak height velocity (PHV) were −0.43 ± 0.24 years, indicating that participants were, on average, close to their estimated PHV. The participants had accumulated an average of 4.3 ± 0.6 years of soccer training experience. According to the training and performance caliber framework proposed by McKay et al. [26], the study population was categorized as Tier 1 (national-level, trained/developmental) athletes. During the 1–2 years preceding the study, the players regularly participated in soccer-specific training sessions 4–5 times per week. Participants continued to engage in team training sessions and official matches throughout the study period. Inclusion criteria required players to be free from injury and to participate consistently in both training and competitive activities. Before participation, players and their parents or legal representatives received written information regarding the potential benefits and risks of the study. Written informed consent was obtained from both parents/legal representatives and athletes after a detailed explanation of the study objectives, procedures, potential risks, and benefits. This study was conducted in accordance with the principles of the latest version of the Declaration of Helsinki. The study protocol was approved by the Local Ethics Committee of the National Centre of Medicine and Science of Sports, Tunis (approval number: CNMSS-LR09SEP01) on 15 September 2023, prior to the commencement of the study. None of the participating athletes had a history of psychological and/or musculoskeletal, neurological, or orthopedic disorders six months prior to the start of the study.

### 2.3. Testing Procedures

All assessments were conducted under similar environmental conditions and at the same time of day to minimize circadian influences on performance. Participants were instructed to avoid strenuous exercise during the 24 h preceding testing and to maintain their normal dietary and hydration habits. Before testing, players completed a standardized warm-up consisting of light running, dynamic stretching, mobility exercises, and progressive sprint drills [19]. The testing sequence was standardized as follows: anthropometric assessment, jump testing, sprint testing, maximal aerobic speed assessment, and repeated-sprint sets testing. Adequate recovery periods were provided between tests to minimize fatigue effects [19].

#### 2.3.1. Anthropometric Assessment

Body height and body mass were measured using a wall-mounted stadiometer (Seca, Hamburg, Germany) and a calibrated electronic scale (Baty International, Burgess Hill, UK), respectively. Body composition was assessed through the measurement of skinfold thickness using Harpenden calipers, and the sum of skinfolds was used for subsequent analyses. Anthropometric assessments were performed following the procedures described by Deurenberg et al. [20], who demonstrated comparable prediction accuracy of body composition estimates in adolescent and adult populations. Biological maturation status was subsequently estimated using the maturity offset method proposed by Mirwald et al. [27]. This approach provides an estimation of the individual’s timing relative to peak height velocity (PHV), which represents the period of maximal growth in stature during adolescence and is considered an indicator of somatic maturation. The maturity offset was calculated by estimating the number of years before or after PHV based on anthropometric characteristics and chronological age. The Mirwald equation has previously demonstrated acceptable validity, with standard errors of estimate of 0.57 and 0.59 years, respectively [27].

#### 2.3.2. Jump Performance Assessment

Lower-body explosive power was assessed through a battery of vertical and horizontal jump tests, administered using a standardized protocol.

##### Vertical Jump Tests

Vertical jump performance was evaluated using three tests: the squat jump (SJ), the countermovement jump, and a 15 s repeated-jump test. Jump height was estimated to the nearest 0.1 cm from flight time, recorded via an infrared timing system (Optojump; Microgate, Bolzano, Italy), which has demonstrated excellent concurrent validity with force platforms (Intraclass correlation coefficient (ICC) = 0.99, 95% confidence interval [CI]: 0.97–0.99) [28].

##### Countermovement Jump

Participants began in an upright standing position with hands fixed on the hips to eliminate any arm-swing contribution. From a fully extended position, they performed a rapid downward movement until reaching approximately 90° knee flexion, immediately followed by a maximal vertical jump. The ICC for the countermovement jump was 0.97 (95% CI: 0.93–0.99) [29].

##### Squat Jump

Participants began in an upright position with knees and hips fully extended and hands placed on the hips throughout the movement. They then performed a controlled downward eccentric action to approximately 90° knee flexion and held this position for ~2 s before executing a maximal concentric vertical jump, without any preparatory countermovement. The ICC for the squat jump was 0.95 (95% CI: 0.89–0.98) [29]. For both tests, the better performance across two trials was retained for statistical analysis.

##### 15 s Repeated Jump Test

Participants performed consecutive maximal vertical jumps during a 15 s period, with hands on hips and knees fully extended between contacts. Total jump height and mean jump power were recorded. The reliability of this test has been previously established in elite youth soccer players (ICC = 0.91, SEM = 0.30) [30], with jump height estimated from flight time via an infrared timing system.

#### 2.3.3. Horizontal Jump Tests

Horizontal explosive power was assessed using the five-jump test and the standing long jump. Both tests have demonstrated good validity and reliability for assessing lower-limb explosive power in soccer players [31].

##### Five-Jump Test

Participants performed five consecutive maximal forward bounds from a stationary standing start, alternating left and right foot contacts, with feet together at takeoff and landing. Arm swing was permitted to facilitate power generation. Trials were repeated if the participant fell backward or landed on a single foot on the final stride. Total distance was measured to the nearest centimeter using a tape measure. The five-jump test has demonstrated good test–retest reliability in elite soccer players (ICC = 0.91; CV = 2.2%) [32].

##### Standing Long Jump

Participants stood behind a marked take-off line with their feet approximately shoulder-width apart. From a stationary position, they performed a rapid countermovement of the lower limbs accompanied by an arm swing and jumped forward as far as possible. The distance from the take-off line to the nearest heel contact upon landing was measured to the nearest centimeter. Participants completed three trials, with the best performance retained for analysis. The standing long jump demonstrated excellent test–retest reliability (ICC = 0.97, 95% CI: 0.93–0.99) [28,33].

#### 2.3.4. Sprint Performance

Sprint performance was assessed via a 30 m sprint with split times at 10 m and 30 m recorded using infrared timing gates (Brower Timing System, Salt Lake City, UT, USA; accuracy: 0.01 s) placed approximately 0.75 m above the surface. Relative reliability of split-time sprint assessment using timing gates in academy male adolescent soccer players ranged from moderate to excellent (ICC = 0.53–0.93), improving with increased sprint distance [34]. Maximal sprint performance was derived from the flying 20 m phase (10–30 m), with an ICC of 0.90 previously reported for this measure [34]. Two maximal trials were performed from a standing start with ≥3 min passive recovery; the best trial was retained for analysis.

#### 2.3.5. Maximal Aerobic Capacity

Maximal aerobic capacity was assessed using the Vam-Eval test, a modified version of the Université de Montréal Track Test [35]. This field-based incremental running test has been validated as a reliable method for estimating maximal aerobic speed and predicting oxygen uptake (estimated VO_2_max) in soccer players. The protocol commenced at an initial running speed of 8.5 km·h^−1^, with increments of 0.5 km·h^−1^ per minute until volitional exhaustion. Running pace was regulated by auditory signals corresponding to 20 m intervals delineated by marker cones around a 200 m indoor athletics track. The test was terminated when a player failed to reach the next cone marker at the required time on two consecutive occasions. Maximal aerobic speed was defined as the running velocity of the last fully completed stage and recorded in km·h^−1^. Estimated VO_2_max was subsequently calculated from the maximal aerobic speed obtained during the Vam-Eval test using the established prediction equation. The typical error of measurement for the Vam-Eval test, expressed as a coefficient of variation (CV), was 3.5% (90% CI: 3.0–4.1%) [36]. Heart rate was monitored continuously throughout the test using a telemetric heart rate monitoring system (Polar Electro Oy, Kempele, Finland), and peak heart rate was recorded as the highest value attained. Rating of perceived exertion (RPE) was assessed immediately at test termination using the Borg CR-10 scale [37].

#### 2.3.6. Repeated-Sprint Sets Test

The repeated-sprint sets protocol comprised two sets of 5 × 20 m sprints, with 15 s of passive recovery between sprints and 1 min between sets [30]. Following each sprint, participants performed a 10 m deceleration phase and a 10 m active jog recovery prior to the next effort. Sprint times were recorded using a photocell timing system (Brower Timing System, Salt Lake City, UT, USA; accuracy: 0.01 s) mounted at a height of approximately 0.75 m above the surface. All participants adopted a standing start with the front foot positioned 0.5 m behind the first timing gate and were instructed to sprint maximally over the full 20 m distance.

Three performance indices were derived for each individual set (5 sprints) as well as across both sets combined. For each set, the following variables were computed: the best sprint time, the total sprint time, defined as the sum of the five sprint times within the set, and the fatigue index, calculated according to the equation proposed by Fitzsimons [38]. The fatigue index represents the percentage decline in sprint performance across repeated efforts and was calculated as [(total sprint time ÷ ideal sprint time) × 100] − 100, where ideal sprint time corresponds to the fastest sprint time multiplied by the number of sprints performed. Across the two sets combined, the sum of all sprint times was additionally recorded as a global performance index. The ICC (95% CI) and CV for sum of sprint times were 0.93 (0.85–0.97) and 0.37%, respectively, while those for best sprint times were 0.81 (0.57–0.92) and 1.45% [30]. Finger-tip capillary lactate concentrations were measured before and three minutes after repeated sprint sets test using a hand-held Lactate Pro device (Arkray, Kyoto, Japan).

### 2.4. Statistical Analysis

The normality of data distribution was verified using the Shapiro–Wilk test. A one way repeated-measures analysis of variance (ANCOVA) was performed to examine the changes over time for each variable with individual’s timing relative to peak height velocity as covariate. To compare the changes over time over the first and second set of the repeated sprint test, a two-way repeated-measures ANOVA was performed. When significant interactions or main effects were detected, Holm-Bonferroni post hoc tests were applied to identify pairwise differences. Effect size was evaluated with partial eta squared (η_p_^2^) where 0.01 < η_p_^2^ < 0.06 constituted a small effect, 0.06 < η_p_^2^ < 0.14 a medium effect, and η_p_^2^ > 0.14 a large effect [39]. The level of significance was set at *p* < 0.05, and all data are expressed as mean ± standard deviation (SD). Statistical analysis was performed in JASP 0.97.1 (University of Amsterdam, Amsterdam, The Netherlands).

## 3. Results

Anthropometrics changed significantly each time it was tested for all three parameters (F ≥ 12.0, *p* < 0.001, η_p_^2^ ≥ 0.32), with no significant Time*PHV effect (F ≤ 0.21, *p* ≥ 0.806, η_p_^2^ < 0.01). However, a significant effect of the covariate PHV was found for height (F = 127.5, *p* < 0.001) and body mass (F = 15.15, *p* < 0.001). Post hoc comparison revealed that height and body mass significantly increased each time, while body fat decreased (Table 1, Supplementary Materials).

**Figure 1:**
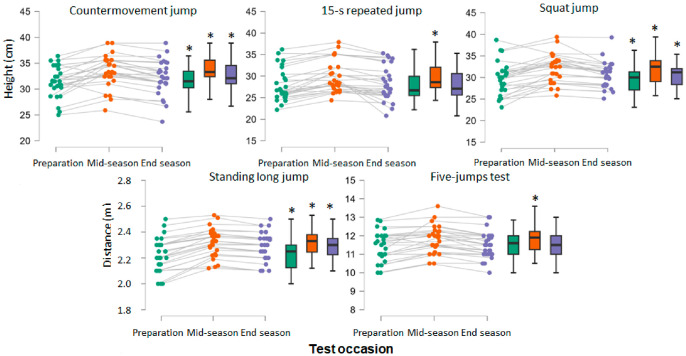
Individual values (left panels; green = preparation, orange = mid-season, purple = end season) and box-and-whisker plots (right panels) showing horizontal and vertical jump performance across the three testing occasions during the season. Box plots represent the median, interquartile range, and minimum–maximum values. * indicates a significant difference compared with all other testing occasions for the respective parameter. 10 and 30 m sprint performances were significantly affected by test occasion (F ≥ 4.12, *p* ≤ 0.022, η_p_^2^ ≥ 0.14), while 20 m flying did not (F = 1.03, *p* = 0.363, η_p_^2^ = 0.04). No significant time*PHV (F ≤ 2.69, *p* ≥ 0.077, η_p_^2^ ≤ 0.10) nor PHV effects (*p* ≥ 0.608) were found for these variables. Post hoc comparison showed that from preparation to mid-season 10 and 30 m sprint performances increased significantly. From mid-season to end season sprint performance at 10 and 30 m decreased again significantly but were still better than at preparation, while flying 20 m did not change significantly (Figure 2, Table S2, Supplementary Materials).

Squat jump, 15 s jump test, and standing long jump were significantly affected by test occasion (F ≥ 4.23, ≤ 0.020, η_p_^2^ ≥ 0.14), while CMJ (*p* = 0.075) and five-jump tests (*p* = 0.052) did not reach significance. No significant Time*PHV interaction effect was found (F ≤ 1.34, *p* ≥ 0.272, η_p_^2^ ≤ 0.05), but a significant PHV effect was found (F = 7.97, *p* = 0.009). Post hoc comparison revealed that all in all jump tests performance significantly increased from preparation to mid-season and decreased again from mid-season to end season. The performance of 15 s repeated jump test and five-jumps test decreased from mid- to end season back to the same level as preparation, while the other test decreased over the same period but jump performance was still better than at preparation (Figure 1, Table S2, Supplementary Materials).

**Figure 2:**
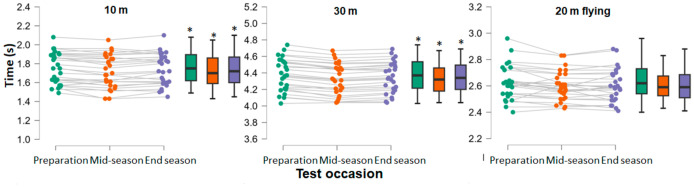
Individual and mean ± 95% CI of sprint times at 10, 20, and 30 m at different time points during the season. Green, orange, and purple colors represent the preparation, mid-season, and end-season testing occasions, respectively. The grey lines indicate individual changes across the three time points. * indicates a significant difference compared with all other time occasions for the corresponding sprint parameter. Estimated maximal aerobic capacity parameters also changed significantly during season (F ≥ 9.00, *p* ≤ 0.002, η_p_^2^ ≥ 0.27). Moreover, a significant time*PHV (F ≥ 4.48, *p* ≤ 0.016, η_p_^2^ ≥ 0.15) and PHV effect (F ≥ 4.32, *p* ≤ 0.048) was found for these variables. RPE (8.7–9.2) during the tests did not significantly change over season (F = 0.75, *p* = 0.479, η_p_^2^ = 0.03) together with maximal heart rate (F = 2.66, *p* = 0.080, η_p_^2^ = 0.10) and no significant interaction (F ≤ 0.27, *p* ≥ 0.76, η_p_^2^ < 0.01) nor PHV effects (*p* > 0.05). Post hoc comparison revealed that VO_2_ max and maximal aerobic speed significantly increased from preparation to mid-season, after which it was kept constant (Figure 3, Table S2, Supplementary Materials).

**Figure 3:**
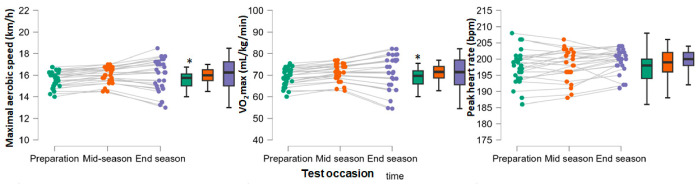
Individual values (left panels) and mean ± 95% confidence intervals (right panels) of maximal aerobic speed (MAS), VO_2_max, and peak heart rate (HRpeak) measured during the tests at the preparation, mid-season, and end-season assessments. Green symbols and boxplots represent the preparation assessment, orange symbols and boxplots represent the mid-season assessment, and purple symbols and boxplots represent the end-season assessment. * indicates a significant difference compared with all other assessment occasions for the corresponding parameter. Regarding the repeated-sprint sets test, a significant effect on total time, best time and fatigue index was found over the season (F ≥ 3.28, *p* ≤ 0.046, η_p_^2^ ≥ 0.11), while lactate produced during tests did not reach significance level (F = 3.00, *p* = 0.059, η_p_^2^ = 0.11). However, significant Time*PHV effects were also found for total and best times (F ≥ 3.50, *p* ≤ 0.038, η_p_^2^ ≥ 0.12, while a significant PHV effect was found for lactate concentration (F = 5.55, *p* = 0.027). Post hoc comparison showed that total sum of sprint times was significantly lower at the mid-season test, while lactate was significantly higher at the end of season test (Figure 4, Table S2, Supplementary Materials).

**Figure 5:**
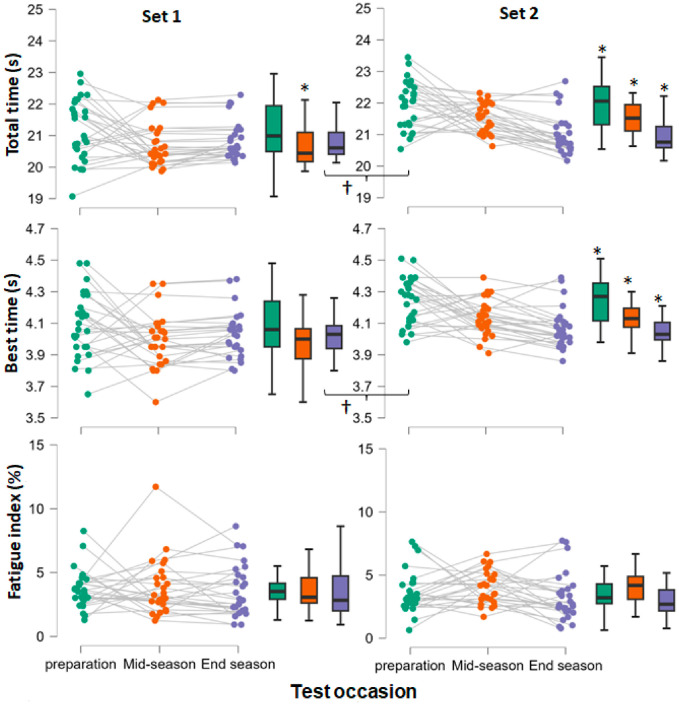
Individual and mean ± 95% CI of repeated-sprint sets test parameters per set at different times during season (teal = preparation; orange = mid-season; purple = end-season). * indicates a significant difference with all other time occasions for this parameter. † indicates a significant difference in time between the first and second set for this parameter at each test occasion.

When evaluating the repeated-sprint sets performance over the two sets a significant effect of testing time (F ≥ 8.26, *p* ≤ 0.001, η_p_^2^ ≥ 0.25), sets (F ≥ 10.74, *p* ≤ 0.003, η_p_^2^ ≥ 0.30) and time*sets interaction effect (F ≥ 3.52, *p* ≤ 0.0037, η_p_^2^ ≥ 0.12) were found for total time and fastest sprint, while no significant effect was found for fatigue index (F ≤ 0.84, *p* ≥ 0.36, η_p_^2^ ≤ 0.03). Furthermore, a significant Time*PHV effect was found for total and best time (F ≥ 3.20, *p* ≤ 0.048, η_p_^2^ ≥ 0.12). No other significant PHV interaction effects were observed (F ≤ 2.27, *p* ≥ 0.145, η_p_^2^ ≤ 0.08) for these variables. Post hoc comparisons showed that in set two all the time parameters decreased significantly each testing time during season, while for set one only the total sprint time was significantly lower at the mid-season test compared with the other two tests, causing the interaction effect. Furthermore, were all time parameters in the first set were always faster than they were in the second repeated sprint set (Figure 5, Table S2, Supplementary Materials).

## 4. Discussion

The present study investigated seasonal changes in anthropometric characteristics, neuromuscular performance, sprint ability, estimated maximal aerobic capacity, and repeated-sprint performance in competitive youth soccer players across the preparation, mid-season, and end-season phases. The main findings revealed progressive increases in body mass and height accompanied by a continuous reduction in body fat percentage, a non-linear pattern of neuromuscular performance characterized by improvements at mid-season followed by partial declines toward the end of the season, significant improvements in estimated aerobic capacity that were maintained after mid-season, and set-dependent adaptations in repeated-sprint performance. Furthermore, biological maturation, assessed through peak height velocity (PHV), significantly influenced several anthropometric and performance variables, highlighting the importance of considering maturational status when interpreting seasonal changes in adolescent athletes.

The progressive increases in body mass and height together with the reduction in body fat percentage reflect the combined influence of biological maturation and regular soccer training. Similar longitudinal changes have been reported in adolescent soccer players, in whom normal somatic growth occurs simultaneously with favorable training-induced adaptations in body composition [14,15]. In the present study, PHV significantly influenced body mass and height but not body fat percentage, confirming that normal growth contributed substantially to changes in body dimensions, whereas the reduction in adiposity was more likely associated with the high energy expenditure of regular training and match participation [12,40]. Because adolescence is characterized by rapid morphological changes that may temporarily influence movement coordination and neuromuscular function [12,13], seasonal changes in physical performance should be interpreted as the combined consequence of maturation and training rather than training adaptations alone.

Jump performance followed a non-linear seasonal pattern, with significant improvements in squat jump, standing long jump, and the 15 s repeated jump test from preparation to mid-season, followed by partial declines toward the end of the season. Nevertheless, squat jump and standing long jump performances remained above baseline values, whereas repeated-jump performance returned to preparation levels. In contrast, countermovement jump and five-jump test performance did not change significantly across the season. These findings agree with previous longitudinal studies reporting that neuromuscular performance typically peaks during the early competitive season following preseason conditioning before partially declining later in the season as training priorities change [41,42]. The mid-season improvements likely reflect enhanced neuromuscular function and movement efficiency resulting from regular training exposure. Conversely, the subsequent decline may be related to accumulated competitive demands, modifications in training content, or maturation-related changes. However, because internal and external training loads were not monitored, the relative contribution of these factors cannot be determined [43]. Importantly, the significant effect of PHV on jump performance further suggests that biological maturation contributed to the observed inter-individual differences in neuromuscular development throughout the season.

Sprint performance demonstrated a similar seasonal pattern. Performance over 10 m and 30 m improved significantly from preparation to mid-season before declining slightly toward the end of the season, although remaining superior to baseline values. In contrast, flying 20 m sprint performance did not change significantly across the competitive season. These findings suggest that acceleration capacity is more responsive to seasonal training adaptations than maximal sprint velocity. Similar observations have been reported in youth soccer players, where improvements in acceleration are commonly achieved during the early competitive season and subsequently maintained despite increasing competitive demands [44,45]. Interestingly, no significant PHV effect was observed for sprint performance, indicating that seasonal variations in sprint ability were relatively independent of maturational status within the present sample.

Estimated maximal aerobic capacity, reflected by maximal aerobic speed and estimated VO_2_max, improved significantly from preparation to mid-season and remained stable thereafter. This pattern is consistent with the expected adaptations following structured preseason and early-season conditioning, during which aerobic fitness is commonly emphasized [46]. The subsequent maintenance of aerobic performance likely reflects sufficient training stimulus to preserve previously acquired adaptations while accommodating the increasing demands of competition [14]. Notably, both maximal aerobic speed and estimated VO_2_max demonstrated significant Time × PHV interaction effects as well as significant PHV effects, indicating that maturation influenced the magnitude of aerobic development across the season. In contrast, maximal heart rate and rating of perceived exertion remained unchanged throughout the season, suggesting that improvements in aerobic performance occurred without detectable alterations in maximal cardiovascular responses or perceived exercise intensity during testing.

Repeated-sprint performance exhibited a more complex pattern of seasonal adaptation. Total sprint time and best sprint time improved significantly at mid-season, whereas blood lactate concentration reached its highest values at the end of the season. Analysis of repeated-sprint performance by set further revealed significant Time × Set interaction effects. Specifically, performance during the first set showed only transient improvements at mid-season, whereas the second set demonstrated progressive improvements across the competitive season. These findings suggest that seasonal adaptations became more evident under conditions of greater physiological stress, when recovery capacity between repeated high-intensity efforts plays a larger role in performance [22]. The significant Time × PHV interactions observed for total and best sprint times further indicate that biological maturation influenced the development of repeated-sprint ability during the season. Moreover, the significant PHV effect on blood lactate concentration suggests that maturation may contribute to inter-individual differences in metabolic responses during repeated high-intensity exercise. Nevertheless, because objective measures of training load, recovery status, and physiological stress were not collected, the mechanisms underlying these adaptations remain speculative. Importantly, fatigue index did not change significantly across the season, indicating that despite improvements in sprint performance, players maintained a relatively stable resistance to fatigue.

The present study has several limitations that should be acknowledged. First, although the longitudinal design with three assessment points provides valuable insight into seasonal changes, the absence of a non-training control group limits the ability to distinguish training-induced adaptations from normal growth and maturation. Second, biological maturation was estimated using the maturity offset equation proposed by Mirwald et al. [35], which provides an indirect estimate of years from peak height velocity rather than a direct assessment of biological age. Although PHV was included as a covariate in the statistical analyses, more precise methods such as skeletal age assessment would further strengthen future investigations. Third, external and internal training-load variables, including GPS-derived match demands, session-RPE during training, training volume, and recovery status, were not monitored, limiting the ability to explain the physiological mechanisms underlying the observed seasonal changes. Finally, participants were recruited from a single competitive team, which may limit the generalizability of the findings to other competitive levels and developmental contexts.

Future studies should include larger multi-team cohorts, non-training control groups, and more frequent longitudinal assessments while integrating objective measures of internal and external training load. In addition, incorporating maturation as a time-varying factor within longitudinal statistical models and combining physical performance assessments with match-performance indicators would provide a more comprehensive understanding of the interactions among biological maturation, training exposure, competitive demands, and athletic development throughout adolescence.

## 5. Conclusions

The present study demonstrated that youth soccer players exhibit significant seasonal variations in anthropometric characteristics and physical performance across a competitive season. Body mass and height increased progressively, while body fat percentage decreased, reflecting the combined influence of growth, maturation, and training exposure. Physical performance followed a non-linear pattern, with improvements in jump, sprint, and aerobic performance from preparation to mid-season, followed by partial declines toward the end of the season. Repeated-sprint performance showed phase-dependent variations, with differences between repeated sets highlighting the complexity of maintaining high-intensity performance throughout a competitive calendar.

From a practical perspective, these findings emphasize the importance of continuous performance monitoring rather than relying solely on pre- and post-season assessments. The mid-season period appeared to represent a phase of enhanced neuromuscular and sprint performance, suggesting a potential window of optimal physical readiness. The subsequent decline observed toward the end of the season may be associated with several interacting factors, including accumulated fatigue, variations in training volume, changes in periodization, and maturation-related processes; however, these mechanisms remain hypothetical because training and match loads were not objectively monitored in the present study. Therefore, future investigations incorporating detailed internal and external load monitoring are needed to better determine the factors contributing to seasonal performance fluctuations. Practically, coaches and practitioners should consider individualized monitoring and adaptive periodized strategies to optimize recovery, maintain neuromuscular qualities, and support performance throughout the competitive season.

## Figures and Tables

**Figure 4:**
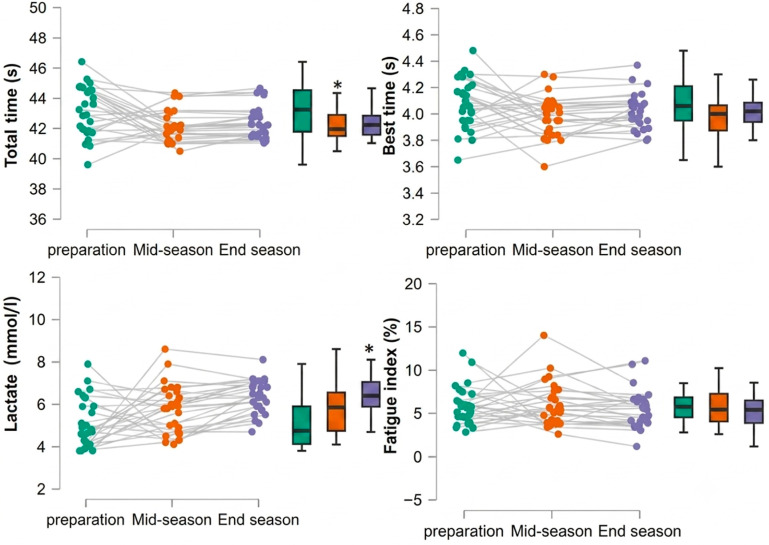
Individual and mean ± 95% CI of repeated-sprint sets test parameters at different times during season (preparation, teal; mid-season, orange; end season, purple). * indicates a significant difference with all other time occasions for this parameter.

**Table 1:** Anthropometric characteristics at different phases of the competitive season.

Variable	Preparation	Mid-Season	End-Season
Body mass (kg)	64.1 ± 6.7	65.6 ± 6.8	66.7 ± 6.8
Height (m)	1.69 ± 0.07	1.71 ± 0.07	1.72 ± 0.07
Body fat (%)	11.3 ± 2.0	10.2 ± 1.8	9.4 ± 1.8

The parameters changed each test time significantly from the others on a *p* < 0.05 level.

## Data Availability

The raw data supporting the conclusions of this article will be made available by the authors upon reasonable request, subject to institutional ethics approval and participant confidentiality requirements, which restrict public sharing of the dataset.

## Supplementary Materials

The following supporting information can be downloaded at: https://www.mdpi.com/article/10.3390/sports14070308/s1, Table S1: Mean ± SD performances with ANCOVA statistics; Table S2: Mean ± SD performances with ANCOVA statistics per set of repeated sprint test.
