# Supplementary material for: Longitudinal Seasonal Development of Anthropometric and Physical Performance Characteristics in Youth Trained Soccer Players
Source: Sports (Basel). 2026 Jul 20;14(7):308. doi: 10.3390/sports14070308 (PMC13417309; doi:10.3390/sports14070308)
Supplement: Supplementary file 1 [file sports-14-00308-s001.zip › sports-4404743-supplementary.pdf]

Table S1. Mean±SD performances with ANCOVA statistics

Variable	*Preparation*	*Mid-season*	*End-season*	*Time effect*			*Interaction effect*			*PHV effect*	
				F	*p*	η^2	*F*	*p*	η^2	*F*	*p*
Body mass (kg)	64.1 ± 6.7	65.6 ± 6.8	66.7 ± 6.8	12.03	< 0.001*	0.32	0.10	0.909	<0.01	15.15	< 0.001*
Height (m)	1.69 ± 0.07	1.71 ± 0.07	1.72 ± 0.07	18.75	< 0.001*	0.43	0.21	0.806	<0.01	127.5	< 0.001*
Body fat (%)	11.3 ± 2.0	10.2 ± 1.8	9.4 ± 1.8	19.09	< 0.001*	0.43	0.16	0.853	<0.01	0.01	0.918
CMJ (m)	31.4±3.0	33.5±3.1	32.2±3.4	2.73	0.075	0.10	0.07	0.929	<0.01	0.29	0.594
Squat jump (cm)	29.6±3.8	32.0±3.3	31.4±3.0	4.23	0.020*	0.14	0.07	0.934	<0.01	1.05	0.316
15 s. jump	27.9±3.8	30.0±3.7	28.2±4.0	7.05	0.002*	0.22	0.76	0.474	0.03	7.98	0.009*
Standing long jump (m)	2.22±0.14	2.32±0.11	2.29±0.11	22.31	< 0.001*	0.47	1.34	0.272	0.05	2.96	0.098
5JT (m)	11.46±0.79	11.79±0.74	11.50±0.72	3.14	0.052	0.11	0.22	0.806	<0.01	3.50	0.073
10m sprint (s)	1.75±0.16	1.72±0.17	1.74±0.17	4.12	0.022*	0.14	2.69	0.077	0.10	0.27	0.608
20m flying (s)	2.63±0.13	2.60±0.11	2.60±0.11	1.03	0.363	0.04	0.14	0.87	<0.01	0.13	0.722
30m sprint (s)	4.38±0.19	4.33±0.19	4.35±0.19	12.18	< 0.001*	0.33	1.54	0.225	0.06	0.25	0.623
Maximal aerobic speed (km/h)	15.6±0.7	16.0±0.7	16.1±1.5	9.20	< 0.001*	0.27	4.48	0.016*	0.15	4.32	0.048*
VO_2 max	68.9±4.1	71.2±4.1	71.2±5.2	9.29	< 0.001*	0.27	5.51	0.007*	0.18	4.53	0.043*
Heart rate (bpm)	197.4±5.2	198.3±4.7	199.6±3.7	2.66	0.080	0.10	0.27	0.764	0.01	4.19	0.051
RPE in VO_2 max test	8.67±1.33	8.93±0.78	9.22±0.89	0.75	0.479	0.03	0.06	0.942	<0.01	0.02	0.899
Total time (s)	43,14±1.68	42.20±1.08	42.39±1.04	7.16	0.002*	0.22	3.50	0.038*	0.12	0.38	0.544
Best time (s)	4.08±0.19	3.98±0.15	4.02±0.14	6.94	0.002*	0.22	4.39	0.018*	0.15	0.14	0.709
Lactate (mmol/l)	5.1±1.2	5.8±01.1	6.4±0.8	3.00	0.059	0.11	0.21	0.813	0.01	5.55	0.027*
Fatigue index (%)	5.9±2.2	6.1±2.5	5.5±2.2	3.28	0.046*	0.12	1.18	0.315	0.05	0.07	0.799

* indicates a significant effect on p<0.05 level.

Table S2. Mean±SD performances with ANCOVA statistics per set of repeated sprint test

**Variable**	**Total time (s)**			**Best time (s)**			**Fatigue index (%)**		
	Set 1		Set 2	Set 1		Set 2	Set 1		Set 2
Result	21.18±0.98		21.96±0.76	4.09±0.21		4.24±0.14	3.7±1.5		3.6±1.7
Effect	*F*	*p*	η^2	*F*	*p*	η^2	*F*	*p*	η^2
Time	8.26	< 0.001*	0.25	9.13	< 0.001*	0.27	0.48	0.621	0.02
Time*PHV	3.22	0.048*	0.11	4.20	0.021*	0.14	0.49	0.616	0.02
Set	14.08	<0 .001*	0.36	10.74	0.003*	0.30	0.84	0.367	0.03
Set*PHV	2.27	0.145	0.08	0.46	0.502	0.02	0.90	0.353	0.03
Time*set	3.91	0.026*	0.14	3.52	0.037*	0.12	0.62	0.543	0.02
Time*set*PHV	2.26	0.115	0.08	2.12	0.131	0.08	1.53	0.227	0.06
PHV	0.33	0.572		0.11	0.744		0.12	0.731	

* indicates a significant effect on p<0.05 level.
